# Exploring Middle School Students’ Interactions and Perceptions of Using Generative AI in STEAM Learning Environments

**DOI:** 10.3390/bs16091626

**Published:** 2026-09-11

**Authors:** Qianhui Zhao, Shuhui Li

**Affiliations:** 1 School of Mathematical Sciences, East China Normal University, Shanghai 200241, China; 52275500047@stu.ecnu.edu.cn; 2 Shanghai Institute of AI for Education, Shanghai 200241, China

**Keywords:** generative artificial intelligence, STEAM/STEM, human–machine interaction, secondary school mathematics

## Abstract

Generative artificial intelligence (GenAI) is rapidly reshaping educational practice, yet its role in supporting K-12 STEAM learning remains underexplored. Using an exploratory design, this study aims to implement two GenAI-based STEAM learning environments, exploring middle school students’ interaction behaviors and perceptions of using GenAI across two 120 min modules: Mathematics-Engineering and Mathematics-Arts. Data were collected from 12 eighth-grade students in China through student–GenAI interaction logs and follow-up interviews. The results revealed that the students working in groups mainly used GenAI to acquire STEAM knowledge/information, relied on exact copying of instructions, favored direct retrieval/review prompts, and used GenAI mainly to request information. Comparatively few interactions involved review, verification, evaluation, or reflection. The participants perceived various roles of GenAI and expressed high satisfaction and trust; however, they demonstrated limited critical evaluation of GenAI outputs, lacked a sense of engagement, and achieved minimal feelings of accomplishment. Although the interaction counts were relatively comparable across the two modules, satisfaction was slightly higher in the Mathematics-Engineering module. Most students perceived no module difference in trust and GenAI utility. Given the small sample and short-term two-module design, the findings are exploratory and context-specific, limiting their generalizability and precluding conclusions about the comparative effectiveness of GenAI-supported STEAM learning. This study provides preliminary evidence on how students interact with and perceive GenAI in STEAM contexts and highlights the need for context-sensitive integration, explicit support for critical evaluation, faded prompt scaffolding, and deeper human–AI collaboration.

## 1. Introduction

Generative artificial intelligence (GenAI) has sparked a technological revolution across multiple societal domains, particularly in education (e.g., L. Huang & Luo, 2026; Yusuf et al., 2024). Many countries and international organizations have prioritized the ethical integration of AI in education, gradually fostering a global consensus within the educational community (e.g., Ministry of Education, 2024; OECD, 2023; Office of Educational Technology, 2023). With its outstanding performance in generating highly human-like dialogue, content creation, data analysis, and problem-solving tasks (Akhtar, 2024; Kasneci et al., 2023), GenAI is progressively reshaping educational paradigms by optimizing teaching practices, facilitating personalized learning, and redefining the broader educational experience (El Fathi et al., 2025; Humphry & Fuller, 2023; Leng, 2024). Nevertheless, the adoption of GenAI in education still faces numerous challenges and concerns, and studies examining students’ GenAI use remain limited, particularly in K-12 contexts. The existing literature primarily concentrates on higher education and single-discipline applications, with scant attention to STEAM (Yusuf et al., 2024).

STEAM (Science, Technology, Engineering, Arts, and Mathematics) education plays a crucial role in cultivating future innovators by equipping them with the comprehensive competencies needed to tackle complex real-world challenges (Chiu et al., 2025; L. Ma et al., 2022; Wu et al., 2023). However, traditional STEAM implementation still faces two persistent challenges. First, structural constraints such as limited time and large class sizes often preclude personalized guidance (Wu et al., 2025), resulting in diminished engagement. Second, for students with limited prior STEAM knowledge, rigid disciplinary boundaries and cognitive conflicts may hinder effective knowledge integration (Cai et al., 2024).

GenAI offers promising solutions to these challenges. First, through natural language interactions, GenAI can deliver dynamic, adaptive learning scaffolds (El Fathi et al., 2025) and provide personalized instructional support tailored to individual learners (Kasneci et al., 2023; U. Lee et al., 2024). Second, by leveraging its extensive cross-disciplinary knowledge base, GenAI can compensate for both teachers’ and students’ subject-area knowledge limitations (Iku-Silan et al., 2023), offering real-time support for emergent ideas and complex problems. This fundamentally transforms traditional classrooms that depend on static and pre-defined curriculum resources (Banda & Nzabahimana, 2023; Santilli et al., 2025) to a more open-ended and flexible learning environment. While the diversity and accessibility of GenAI tools bring new opportunities, empirical research examining GenAI applications in K-12 STEAM remains scarce (Marzano, 2026).

Existing studies have primarily examined the learning outcomes and effects of GenAI use (e.g., T. C. Yang et al., 2025), while paying less attention to the micro-level processes through which students interact with GenAI and perceive its role in K-12 STEAM contexts. This gap is important because STEAM learning is typically interdisciplinary and open-ended, requiring students to integrate knowledge, generate ideas, test solutions, and refine artifacts. In such contexts, GenAI may serve not only as an answer provider but also as a knowledge resource, design partner, feedback provider, and creativity scaffold. However, evidence from language learning suggests that students often use GenAI mainly to obtain direct answers, with few follow-up questions or requests for further explanation (Durgungoz & Kharrufa, 2026). Whether similar patterns occur in STEAM tasks remains unclear. Thus, examining both students’ interactions with GenAI and their perceptions of its role is necessary to understand whether and how GenAI can support exploratory, reflective, and knowledge-building engagement in STEAM learning, thereby informing its meaningful integration into K-12 STEAM education.

Accordingly, this study aims to examine how middle school students interact with and perceive GenAI in STEAM learning environments. By comparing student experiences across two modules (mathematics-engineering vs. mathematics-arts), the study seeks to explore how GenAI might be integrated into K-12 STEAM education, offering preliminary implications for instructional implementation of GenAI-enhanced STEAM education for K-12 learners.

## 2. Literature Review

### 2.1. Students’ GenAI Use in School Learning

The diversity and accessibility of GenAI tools offer significant potential to transform educational practices across STEAM disciplines. Current research has predominantly focused on higher education contexts (W. Huang et al., 2025; Schei et al., 2024), targeting undergraduate or graduate students as participants in GenAI interventions. These studies have explored GenAI applications in technology (e.g., A. C. Yang et al., 2024), engineering (e.g., Kim et al., 2024), science (e.g., Tassoti, 2024), mathematics (e.g., Gouia-Zarrad & Gunn, 2024), as well as integrated STEAM (e.g., P.-H. Li et al., 2025).

In contrast, research focusing on K-12 students using GenAI in STEAM disciplines is notably scarce (Marzano, 2026). The extant literature primarily focuses on (1) scientific inquiry (e.g., Ng et al., 2024), (2) programming (e.g., T. C. Yang et al., 2025), (3) mathematical problem-solving (e.g., Cheng et al., 2024), and (4) interdisciplinary integration (e.g., Iku-Silan et al., 2023). These studies mainly employ quasi-experimental designs, using GenAI as an intervention tool to compare with traditional approaches. Findings indicate that GenAI can facilitate conceptual understanding and knowledge construction (El Fathi et al., 2025; J. Lee et al., 2023), improve academic performance, cultivate sustained learning habits, reduce students’ learning anxiety, boost motivation (Ng et al., 2024), enhance critical thinking, creativity, and attitudes toward STEAM disciplines, and effectively address challenges in developing higher-order thinking skills in STEAM education (Šedlbauer et al., 2024; Wu et al., 2025).

However, several concerns have also emerged regarding both students’ misuse of GenAI and current technological limitations. Specifically, GenAI use may foster laziness or career-related anxiety (Yilmaz & Yilmaz, 2023), induce lower flow experience and self-efficacy (T. C. Yang et al., 2025), and reinforce quantitative misconceptions that reveal its limitations in problem-solving and numerical calculation (El Fathi et al., 2025).

As discussed above, most studies adopt a macro-level perspective, focusing on GenAI’s impacts on students’ learning outcomes and affective domains, while largely neglecting micro-level human–GenAI interaction processes. Prior attempts to examine student–GenAI interactions typically classify input questions or prompts, including (1) question content and structure types (J. Lee et al., 2023), (2) information versus review requests (Min et al., 2025), or (3) about 20 prompt types (e.g., exact copy of instruction, partial copy of instruction, and refining GPT code; Güner & Er, 2025). Some studies have extended beyond prompt analysis by incorporating GenAI responses, treating each question-answer pair as the smallest analytical unit. For example, Min et al. (2025) analyzed conversation contents and classified them into variables, materials, experimental processes, analysis, results, and knowledge. Furthermore, researchers have also employed visualization techniques to characterize interaction patterns, with Güner and Er (2025) identifying five student–AI interaction profiles and tracking their evolution using Sankey diagrams, while Min et al. (2025) presented student–AI interactions using an interaction map. Additionally, Zhong et al. (2024) provided a broader perspective on how human–AI interaction unfolds, analyzing participants’ online posts across five dimensions: disciplinary grounding, diversity, cognitive advancement, integration, and reflection.

Taken together, three interrelated gaps remain in research on student–GenAI interaction. First, at the population level, empirical evidence is still concentrated in higher education, while K-12 studies remain limited and often examine single subjects rather than STEAM learning. Second, at the analytical level, existing interaction taxonomies focus on either technology-related features (prompt type/purpose) or on dyadic conversation content, while paying insufficient attention to students’ own discursive standpoints. This omission warrants attention because students’ positioning toward GenAI may shape not only the questions they ask but also the learning opportunities that emerge from the interaction. Recent evidence further suggests that shifts in students’ epistemic stance toward AI, rather than the surface-level features of their prompts alone, may predict subsequent learning gains (Abdelghani et al., 2026). Third, at the methodological level, interaction profiles are often presented qualitatively, with limited quantitative evidence to support comparison across modules, cohorts, or disciplinary combinations. These gaps highlight the need to move beyond fragmented accounts of GenAI use toward a more comprehensive and fine-grained understanding of student–GenAI interactions in STEAM contexts.

### 2.2. Students’ Perception of GenAI Use

Beyond students’ actual interactions with GenAI, their perceptions of GenAI use constitute another important dimension of GenAI-supported learning experiences. Existing research has examined these perceptions mainly from three perspectives: students’ evaluations of their learning experiences, their judgments of GenAI and its outputs, and their understandings of GenAI’s roles and functions.

One major line of research has focused on students’ evaluations of their GenAI-supported learning experiences, including satisfaction (e.g., Iku-Silan et al., 2023), perceived usefulness, perceived ease of use (Sun et al., 2024), learning attitudes, expectations/usage intention (B. Ma et al., 2024), and learning interest (Cheng et al., 2024). These studies suggest that students’ experiences of GenAI-supported learning involve multiple interrelated aspects, with satisfaction serving as a direct indicator of their overall learning experience. For example, Iku-Silan et al. (2023) examined learners’ satisfaction with a decision-guided chatbot-based interdisciplinary learning model. Satisfaction has also been identified as an important predictor of students’ and teachers’ continuance intention to use GenAI tools (Kavitha & Joshith, 2025).

A second line of research concerns how students evaluate GenAI and its outputs, with trust emerging as an increasingly important construct. Trust is particularly relevant because students often rely on GenAI systems for information, feedback, and guidance under conditions of uncertainty (Pitts & Motamedi, 2025). Amoozadeh et al. (2024) suggest that students’ trust in GenAI varies considerably, ranging from distrust and neutrality to trust. As GenAI increasingly participates in learning by generating explanations, feedback, and recommendations, students’ trust in GenAI has become critical for understanding their learning experiences and decision-making during use (Nazaretsky et al., 2025). Nevertheless, research on students’ trust in GenAI remains concentrated in higher education, with comparatively limited evidence from K-12 contexts (Qiu et al., 2025; Ragone et al., 2026).

Third, students’ perceptions of GenAI are also reflected in how they understand GenAI’s functions and roles. Existing studies have identified a range of perceived functions, including providing specialized programming support and general domain services (Sun et al., 2024), generating code and solutions, supporting technical writing, explaining complex concepts, assisting assessment preparation, and enhancing engagement through continuous availability (Singh et al., 2023). GenAI has also been conceptualized as a colleague, teacher, or tool (Min et al., 2025), as well as a guide or consultant (Prakasha et al., 2024). Students recognize GenAI’s ability to deliver interesting and motivating learning experiences (Shoufan, 2023) while providing multifaceted assistance, including explaining programming concepts, offering code examples, answering programming questions, providing learning guidance and resources, and debugging programming code (B. Ma et al., 2024). At the same time, students express concerns about potential risks, such as inaccurate responses, inability to replace human intelligence, potential facilitation of malicious use, possible threats to employment opportunities (Shoufan, 2023), and excessive flexibility with uncontrollable recommendations (Ng et al., 2024).

Taken together, three gaps remain in the literature. First, existing evidence is still concentrated in higher education and single-disciplinary contexts, with limited research involving K-12 students, particularly in interdisciplinary STEAM learning environments. Second, perceptual factors such as satisfaction, trust, usefulness, attitudes, and interest have often been examined separately, resulting in a fragmented understanding of how students experience and evaluate GenAI use. Third, prior studies have mainly focused on students’ overall evaluations, while less is known about how these perceptions change during the learning process and whether they differ across various STEAM contexts. These gaps highlight the need to examine multiple aspects of students’ perceptions and their changes in K-12 STEAM learning environments, as well as comparing how such perceptions manifest across different STEAM modules.

### 2.3. Conceptual Framework

**Figure 1:**
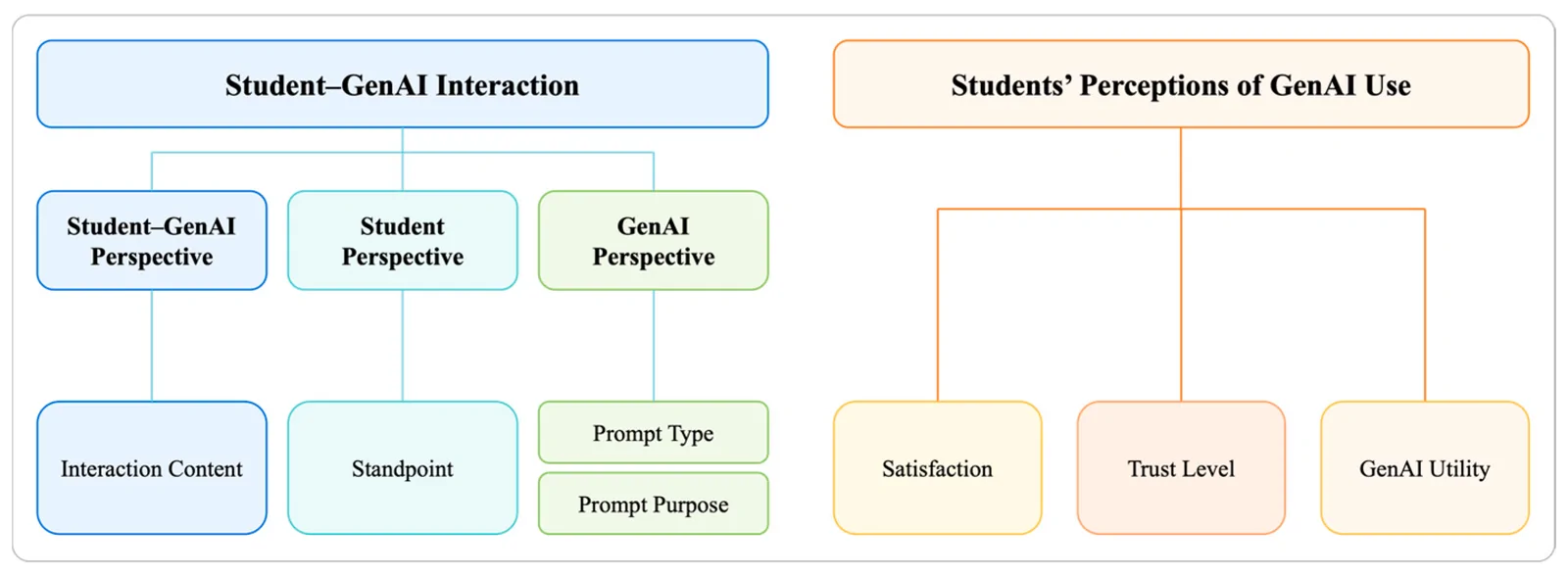
Conceptual framework.

Based on the literature reviewed above, this study developed a conceptual framework comprising two dimensions: student–GenAI interaction and students’ perceptions of GenAI use (see Figure 1). The interaction dimension examines student–GenAI interaction from three perspectives: the “Student–GenAI” perspective, the “Student” perspective, and the “GenAI” perspective. The perception dimension examines students’ perceptions through three sub-dimensions: satisfaction, trust level, and GenAI utility.

For the interaction dimension, this study proposes a framework with four sub-dimensions across three perspectives (see Table 1). From the Student–GenAI perspective, interaction content captures what students discuss with GenAI, such as STEAM knowledge, inquiry plans, and problem-solving processes. From the Student perspective, student standpoint captures how students engage with and respond to instructional and GenAI-generated discourse. This sub-dimension is informed by classroom discourse analysis (Sinclair & Coulthard, 1975) and by research on monologic and dialogic tendencies in children’s discourse (Haworth, 1999). From the GenAI perspective, prompt type describes how students formulate requests to GenAI, while prompt purpose identifies what students seek to achieve through these requests. Together, these sub-dimensions provide complementary evidence of student–GenAI interaction. It should be noted that the same interaction behavior may reflect different cognitive processes across contexts. Therefore, the present framework maintains its interpretation at the behavioral level.

For the perception dimension, this study focuses on satisfaction, trust level, and GenAI utility. These three sub-dimensions were selected because they correspond to students’ learning experience, trustworthiness judgments, and learning value. Their selection is also supported by recent empirical work that jointly examined satisfaction, trust, and utility in students’ perceptions of GenAI use (C. F. Lee et al., 2026).

Specifically, satisfaction was conceptualized following Suchanek and Kralova’s (2025) definition as students’ short-term attitude and overall subjective response to the educational experience of using GenAI. Prior studies have measured satisfaction using multi-item questionnaires (e.g., Iku-Silan et al., 2023; Suchanek & Kralova, 2025) and graphical self-report methods (Satyam et al., 2022). As this study focuses on students’ overall satisfaction, its changes during learning, and the reasons underlying these changes, satisfaction was operationalized through a single retrospective 5-point rating item, supplemented by open-ended questions probing the reasons. Students could also refer to their previously drawn emotion graphs as recall aids when describing changes in satisfaction during the learning process (Satyam et al., 2022).

Trust level was conceptualized based on Pitts and Motamedi’s (2025) definition of student–AI trust as “students’ willingness to rely on AI system’s guidance, feedback, and information under conditions of uncertainty and potential vulnerability”. Although prior studies have commonly used multi-item scales (e.g., Jian et al., 2000; Nazaretsky et al., 2025), single-item trust ratings are also used in applied settings because of their simplicity and suitability for immediate assessment (e.g., Razin & Feigh, 2024; Rieger et al., 2022). Moreover, Rieger et al. (2022) reported consistent patterns between single-item ratings and the scale developed by Jian et al. (2000). Accordingly, this study operationalized trust through a single retrospective rating item, supplemented by open-ended questions exploring students’ reasons for their ratings and their verification and reliance behaviors.

GenAI utility refers to the extent to which students perceive GenAI as enhancing their ability to accomplish learning tasks. It is related to perceived usefulness in the Technology Acceptance Model (Davis, 1989) and to prior conceptualizations of GenAI’s functional roles in education, such as tool, teacher, colleague, guide, or consultant (Min et al., 2025; Prakasha et al., 2024). Given the emerging nature of GenAI use in K-12 STEAM education and the need to identify context-specific roles, this study used open-ended retrospective questions to elicit students’ perceived GenAI roles and their evaluations of these roles.

### 2.4. Research Questions

To gain a more comprehensive understanding of K-12 students’ experiences with GenAI in STEAM learning environments, this study examines two aspects: interaction and perception. It further compares these two aspects across different STEAM modules. Specifically, the study addresses the following research questions:

**RQ1.**  *What characteristics are manifested in student–GenAI interactions in STEAM learning environments?*

**RQ2.**  *How do middle school students perceive GenAI use in STEAM learning environments?*

**RQ3.**  *How do student–GenAI interactions and perceptions of GenAI use differ between mathematics-engineering and mathematics-arts modules?*

## 3. Methods

### 3.1. Participants and Research Design

This study was conducted as part of the GenAI-STEAM Project, a research project initiative exploring the integration of GenAI into STEAM learning environments for secondary school students. As GenAI-supported STEAM learning is still at an early stage of classroom implementation, large-scale studies in regular class-size K-12 settings remain constrained by the current limitations of GenAI tools and by practical classroom conditions, including instructional time, classroom management, task supervision, and students’ readiness to use GenAI appropriately. Accordingly, this study adopted an exploratory research design to characterize middle school students’ interactions with GenAI and their perceptions of GenAI use in STEAM learning environments, rather than using a control group to compare GenAI-supported STEAM learning with traditional instruction.

Twelve eighth-grade students from Zhejiang Province, China, were recruited through convenience sampling, and participation was voluntary. The sample comprised nine boys and three girls aged between 13 and 14 years, all of whom reported little or no prior experience with GenAI. The students (S1–S12) were organized into six learning groups (G1–G6). Informed consent was obtained from both students and their parents before the study commenced.

**Figure 2:**
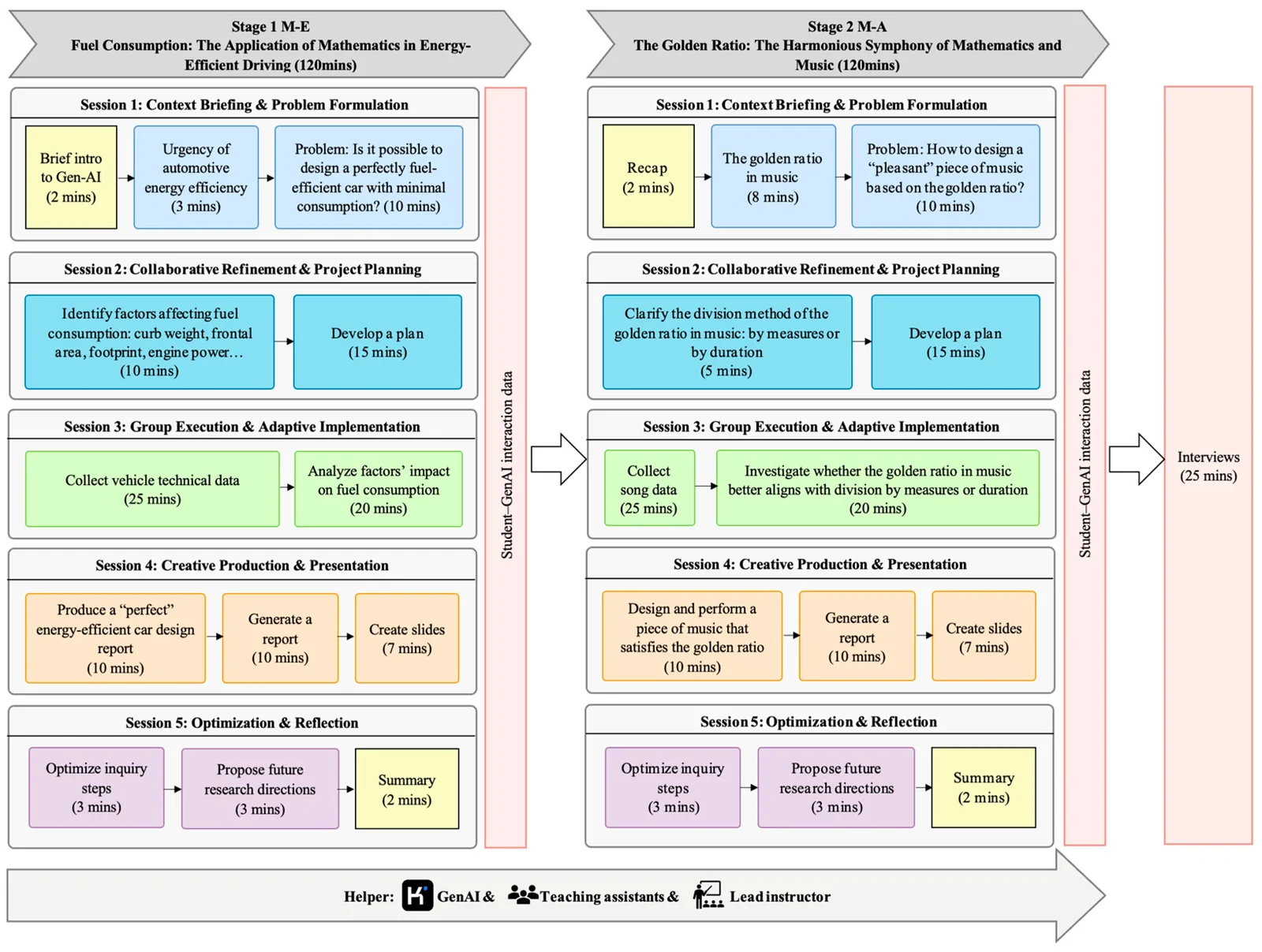
Flowchart.

The intervention was conducted in an informal, quiet off-campus study room in January 2025. Figure 2 shows the flowchart. Each student participated in two 120 min STEAM modules: (1) “Fuel Consumption: The Application of Mathematics in Energy-Efficient Driving” (focusing on mathematics and engineering, M-E), and (2) “The Golden Ratio: The Harmonious Symphony of Mathematics and Music” (emphasizing mathematics and art, M-A). Both modules consisted of five sessions: (1) context briefing & problem formulation, (2) collaborative refinement & project planning, (3) team execution & adaptive implementation, (4) creative production & presentation, and (5) optimization & reflection. Throughout the sessions, each group interacted with GenAI and received scaffolding support from their teaching assistants and the lead instructor. Meanwhile, we continuously recorded student–GenAI interaction logs. Each student was also provided with a blank sheet to briefly track changes in their satisfaction with GenAI use over time by plotting an emotion graph on coordinate axes. Post-intervention, each participant completed a 25 min semi-structured interview, during which they could refer to their emotion graph to help recall their GenAI use experiences.

Each module was led by one instructor, and each group was supported by a teaching assistant. All instructional team members majored in mathematics education, and two had two years of teaching experience. Before the intervention, they received training on module delivery and used the same instructional materials and implementation guidelines. During the intervention, teaching assistants provided on-demand technical support, prompt-formulation guidance, and procedural demonstrations. This support was intended to facilitate students’ interaction with GenAI rather than provide inquiry questions or task solutions.

### 3.2. GenAI Platform Selection

The GenAI platform was selected based on accessibility, platform maturity, and functional suitability for the designed learning activities. Kimi AI was selected because it was readily accessible, relatively stable, and functionally aligned with the two STEAM modules. Its capabilities included multi-turn interaction with contextual continuity, information retrieval and synthesis, and content generation. The slide-generation function was particularly relevant to the creative production and presentation session and was not available in some alternative platforms considered at the time. Using the same primary platform across all six groups also helped maintain consistency in the learning environment and reduce potential variation. Nevertheless, Kimi was not used exclusively; students could use other GenAI tools (e.g., Doubao) and supplementary search engines (e.g., Baidu) for cross-referencing when needed.

Accordingly, one laptop with access to Kimi AI (https://kimi.moonshot.cn/) was provided to each group. The study used Kimi k1 with default model and platform settings, without additional researcher configuration. Within each module, each group generally interacted with GenAI in the same conversation so that the conversational context could be maintained. A new conversation was initiated for the subsequent module. Thus, chat history was not carried over between the mathematics-engineering and mathematics-arts modules. The slide-generation task was an exception because the Kimi+ function automatically opened a separate conversation without retaining previous chat history, thereby interrupting conversational continuity for that task.

### 3.3. Data Collection

Two primary data sources, student–GenAI interaction logs and interviews, were collected to examine students’ interactions with GenAI and their learning experiences. For student–GenAI interaction logs, teaching assistants archived each group’s interactions with GenAI after each session. As students worked collaboratively in pairs and shared one device within each group, the interaction logs represented group-level rather than individual-level interactions. The archived records comprised complete dialogue transcripts, screenshots of key interaction episodes, and links to saved chat sessions. Across the two STEAM modules completed by the six groups, a total of 12 interaction log datasets were obtained.

The interview protocol (see Appendix A) was designed to explore students’ perceptions of GenAI’s use in STEAM learning environments, covering four sub-dimensions: user satisfaction, trust level, perceived utility, and other issues (e.g., advantages/disadvantages, memorable events, and suggestions). For example, in terms of trust level, a sample question is “On a scale of 1–5, how much do you trust the information provided by GenAI? Why?” All 12 interviews were audio-recorded and transcribed for analysis.

### 3.4. Data Coding

Regarding student–GenAI interaction logs, each prompt and its corresponding GenAI response were treated as a single analytical unit, resulting in a total of 310 entries. We named entries (1) in M-E as M-E-1 to M-E-184, and (2) in M-A as M-A-1 to M-A-186. Based on the analytical framework presented in Table 1, two trained coders independently analyzed a stratified random sample (20%, *n* = 62), with Cohen’s κ values of 0.964, 0.975, 0.943, and 1.000 for the sub-dimensions of interaction content, student standpoint, prompt type, and prompt purpose, respectively. Coding discrepancies were resolved through multiple rounds of discussions until consensus was achieved. Then, the primary coder subsequently processed the remaining dataset.

**Figure 3:**
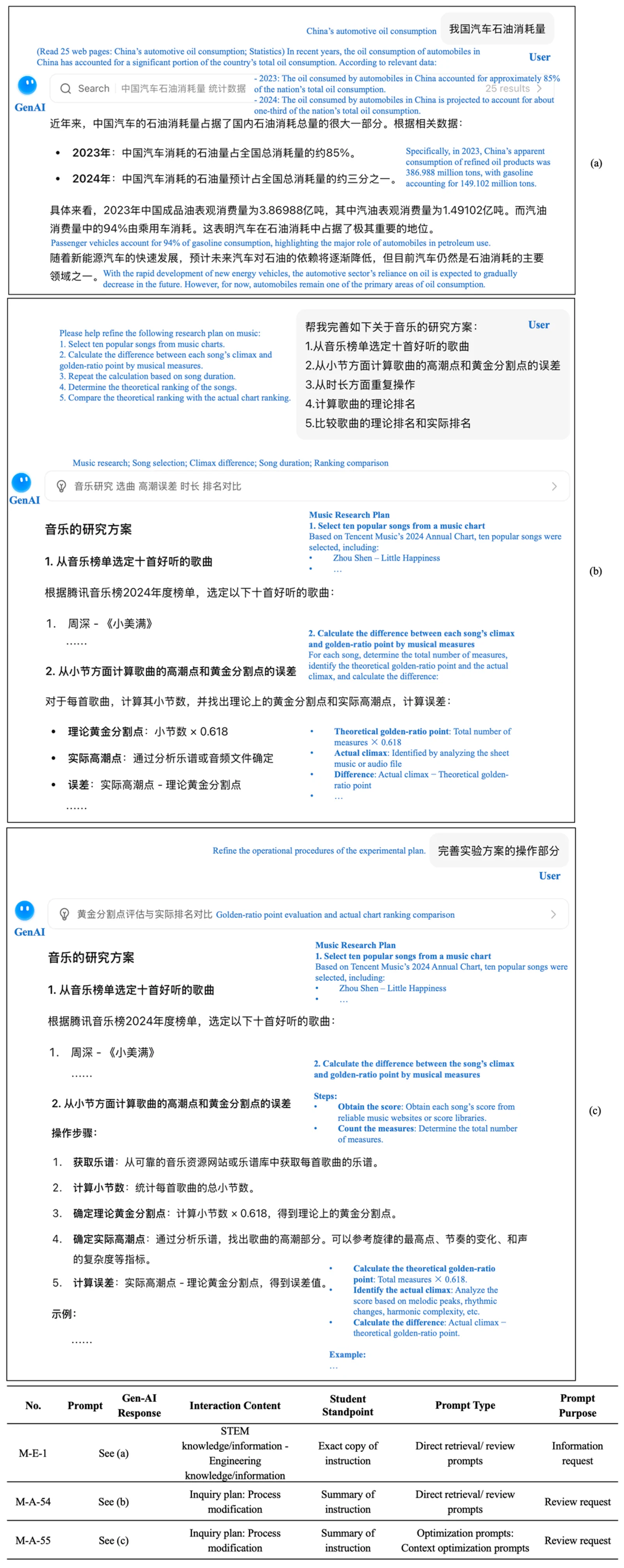
Coding example of student–GenAI interaction log: (**a**) coding sample from G1; (**b**,**c**) two consecutive coding samples from G3.

Figure 3a illustrates a coding sample from G1. G1 entered a direct retrieval prompt (this prompt appeared for the first time, was non-reflective, and fell within GenAI’s functional scope)—namely, exactly copying the instructor’s instruction from session 1 of the M-E module, which was intended to introduce the real-life context and help students recognize the urgency of automobile energy conservation: “Please retrieve China’s annual automobile fuel consumption in recent years”—to request specific engineering knowledge/information from GenAI without personal ideas or perspectives. Therefore, the purpose of this prompt was an information request. Figure 3b and Figure 3c show two consecutive examples from G3, respectively. G3 first entered a direct review prompt by summarizing the instructor’s instruction to “design an inquiry plan to explore the relationship between music and the golden ratio” into the basic steps of an inquiry plan and asking GenAI to refine it as a review request, thereby modifying the inquiry process within the inquiry plan. Subsequently, after receiving GenAI’s response, G3 built on the preceding interaction by further focusing on the operational procedures of the inquiry plan and requesting refinement. Therefore, the follow-up prompt was coded as a context optimization prompt.

In addition, coding for the interaction content sub-dimension required joint analysis of both prompts and GenAI’s responses. GenAI responses were used only to determine interaction content, rather than to evaluate response quality. The remaining sub-dimensions were coded based solely on prompts.

Multiple coding was allowed for the *interaction content* and *prompt type* because their categories were not mutually exclusive. For example, M-E-22 (see Appendix B) was coded as both mathematical model construction and solving and mathematical knowledge/information in the *interaction content*. Similarly, M-E-12 (“Plot the graph of the function”) was coded as both a solution optimization prompt and a non-functional scope prompt in the *prompt type*. In contrast, single coding was applied to the other two sub-dimensions. Additional coding samples are provided in Appendix B.

Interview data were analyzed using a thematic analysis approach (Braun & Clarke, 2006). Two coders first independently reviewed the transcripts and generated initial codes. They then grouped related codes into broader themes, reviewed and refined these themes by examining their internal coherence and relationships, and resolved discrepancies through discussion until full consensus was reached. Finally, a complete set of themes was systematically analyzed.

### 3.5. Data Analysis

To answer research question 1, we counted the frequency of the student–GenAI interaction data across four sub-dimensions: interaction content, student standpoint, prompt type, and prompt purpose, to characterize the distribution of interaction patterns across these sub-dimensions.

To address research question 2, students’ perceptions of GenAI use were analyzed in terms of satisfaction, trust level, and perceived utility. For satisfaction, means and standard deviations were calculated for overall satisfaction scores. Interview data were further analyzed to identify patterns of changes in students’ satisfaction during the STEAM learning process and the reasons underlying these changes. For trust level, the mean and standard deviation were calculated to analyze trust scores, and interview data were used to examine students’ justifications for their ratings, as well as their information-processing behaviors, including how they verified and used GenAI outputs. Concerning perceived utility, all GenAI roles mentioned in the interviews were identified, categorized, and counted. Students’ preferences between GenAI-supported STEAM learning and traditional instruction were also compared. Student–GenAI interaction data were used as complementary evidence to interpret the perception findings.

To address research question 3, module-based comparisons were conducted for both student–GenAI interactions and students’ perceptions. For student–GenAI interactions, the frequencies of each interaction category were calculated and compared across the two modules. For students’ perceptions, satisfaction scores were compared between the two modules, along with module differences in satisfaction-change patterns and their underlying reasons. Module-related differences in trust level and perceived utility were also examined.

## 4. Results

### 4.1. Students’ Interactions with GenAI

***Interaction content:*** As illustrated in Table 2, the majority of interaction content was related to STEAM knowledge/information, accounting for 51.1% of the total. This was followed by problem-solving process (24.9%) and inquiry plan (15.7%). In contrast, prompts addressing analysis and results (4.3%) and presentation and display (3.5%) trailed significantly behind, along with evaluation and reflection, representing merely 0.5% of the total. Specifically, within the STEAM knowledge/information category, engineering knowledge/information occurred more frequently than mathematical knowledge/information in the M-E module (37.0% vs. 7.1%); music knowledge/information was more frequent than mathematical knowledge/information in the M-A module (34.4% vs. 23.7%). This distribution indicates a stronger emphasis on non-mathematical disciplines in group-level inquiries. Furthermore, since the M-E module examined factors affecting vehicle fuel consumption and the M-A module required extensive collection of musical score data, prompts related to variables and data collection appeared more frequently than others. Notably, the complete absence of result verification and future inquiry directions suggests that at the group level, the students primarily used GenAI for immediate information retrieval rather than critical evaluation or longitudinal learning planning.

***Student standpoint:*** According to Table 3, among the 310 prompts, those reflecting an exact copy of instruction were most prevalent (47.1%), which was followed by prompts exhibiting spontaneous inquiry (28.7%) and summary of instruction (21.3%). In contrast, an exact copy of GenAI’s recommendations was the least common (excluding “Others”, the same applies hereafter and will not be repeated), at only 2.3% of the total. Furthermore, two misinterpretations were observed, which have been annotated in Table 3.

***Prompt type:*** Direct retrieval/review prompts were the most prevalent (64.4%), followed by optimization prompts (33.0%), as shown in Table 3. Within optimization prompts, there were more context optimization prompts than solution optimization prompts, indicating that the students working in groups were more inclined to reflect on whether prompts precisely articulated their needs rather than critically evaluating the validity of GenAI-generated responses. Non-functional scope prompts were the least frequent (1.9%).

***Prompt purpose:*** In Table 3, out of 310 prompts, 303 (97.7%) were information requests, whereas only 5 (1.6%) constituted review requests. This suggests limited utilization of GenAI’s review function at the group level, potentially reflecting an incomplete understanding of its capabilities.

***Module differences:*** Direct retrieval/review prompts and spontaneous inquiry were more frequent in the M-A module than in the M-E module. This difference may reflect module-specific information-seeking practices during spontaneous inquiry. In the M-E module, groups tended to use GenAI to identify sources of automotive technical information and then consulted third-party websites for data. In contrast, in the M-A module, groups more often retrieved song and music-score information directly through GenAI, such as “the simplified music score of Zhang, Shaohan’s Cocoon Breaking” (M-A-18). Review requests appeared only in the M-A module, possibly because experience gained in the M-E module helped teams articulate their own ideas and ask GenAI to evaluate them (e.g., M-A-54 in Figure 3b).

By contrast, optimization engagement was more frequent in the M-E module than in the M-A module. One possible explanation is that engineering is not typically taught as a standalone K-12 subject, and students may have been less familiar with its related concepts. Consequently, groups may have relied more on GenAI to request information, contextual explanations, and simplified responses to support comprehension. In addition, non-functional scope prompts were less frequent in the M-A module. This may reflect a carryover effect from the preceding M-E module, in which unsuccessful attempts with such prompts may have discouraged groups from using them in the later module.

### 4.2. Students’ Perception of GenAI Use

***Satisfaction:*** Overall, the 12 students demonstrated high satisfaction with GenAI use in STEAM learning environments (M = 4.75, SD = 0.40). Interview data further revealed that students’ satisfaction changed throughout the learning process, mainly following two patterns: gradual improvement and fluctuation.

Students whose satisfaction gradually increased attributed this change to growing familiarity with GenAI and recognition of its functionalities. For example, S3 referred to “increasing familiarity through use”, while S12 mentioned “discovering its rich functionalities”. Similarly, S11 noted, “I initially doubted GenAI’s artistic capabilities, but its consistent performance gradually enhanced my satisfaction.” These experiences strengthened students’ perceptions of GenAI’s efficiency and convenience. Notably, several students (S1, S4, S6, S9, and S11) reported relatively low initial satisfaction, mainly due to unfamiliarity with GenAI. As S4 explained, “At first, I struggled to phrase prompts effectively for GenAI, leading to a negative experience.” Limited hands-on opportunities also reduced initial satisfaction; for instance, S1 stated that the lecture-based introductory sessions provided few opportunities to interact with GenAI. These challenges, particularly limited prompt-construction skills and insufficient try-out opportunities, may help explain the high frequency of the “exact copy of instruction” standpoint.

Other students described fluctuating satisfaction, often in response to specific GenAI outputs. Declines were mainly associated with overly complex GenAI outputs (S7), repetitive or inaccurate responses (S8, S10), and misinterpretation of prompt intentions that led to irrelevant outputs (S2, S7). For example, S2 explained, “My satisfaction gradually declined when realizing GenAI couldn’t be completely relied upon. For instance, when attempting to retrieve official documentation regarding China’s passenger vehicle fuel consumption standards (M-E-19 & M-E-23), Kimi provided incomplete files, forcing us to find them via the Baidu search engine.” Other negative experiences included incorrect musical notation with inaccurate bar counts (S7, S8) and contextual confusion caused by Kimi’s memory across consecutive queries (S1). However, satisfaction often recovered when students used GenAI’s slide-generation function. S7 commented that the system could transform text drafts into polished slides with better aesthetics and efficiency than manual production. Some students, such as S2, reported multiple fluctuations but noted that later positive or negative experiences had a weaker impact than earlier ones, suggesting that accumulated experience may have moderated changes in satisfaction.

***Trust level:*** Generally, S1–S12 exhibited a relatively high level of trust in GenAI, with a mean (SD) trust score of 3.91 (0.72). Specifically, three students gave scores of 4.9 or higher, stating that “for every conversation, Kimi displays the number of webpages it has read before responding (typically several dozen), synthesizing various perspectives from the internet” (S4), and “unlike information from a single platform, it is less likely to be biased, so it should be fairly reliable” (S5). As a result, they demonstrated extremely high trust in GenAI, relying “solely on existing knowledge and subjective perception to judge accuracy” (S3). S8, S9, S11, and S12 were all assigned a trust score of 4, citing instances where GenAI provided clearly incorrect information or distracting recommended resources. Some admitted that they “usually do not verify the accuracy but directly adopt key information” (S8) or “would refine the prompts and ask again when faced with unsatisfactory responses from GenAI” (S11, S12), which might explain the relatively low number of solution optimization prompts. Five students gave scores of 3.5 or lower, critically evaluating the GenAI output. For example, S1 questioned the direct source of GenAI’s output, initially believing that it “came from a pre-existing database similar to a question bank rather than an aggregation of online resources”. S2 argued that “GenAI’s information sources also include many false claims from the internet, and when encountering them, I would raise questions to prompt corrections”, leading them to “always remain cautious and, when necessary, turn to third-party search engines or click on source links for verification” (S10).

***GenAI utility:*** The students mentioned 8 distinct roles of GenAI, 4 positive and 4 neutral (see Table 4). Information retrieval/integration assistant and slide generation were mentioned by every student. All students highly recognized GenAI’s extremely rapid process of reading, processing, and outputting information. They noted that it “ultimately presents search results in summarized text” (S2, S10), “along with original webpage references for further click-through verification” (S7). It also “autonomously collects large amounts of data in a short time” (S4) or “enables personalized exploration, which traditional courses cannot achieve” (S5).

Regarding neutral evaluations, students acknowledged GenAI’s assistance in “supplementing missing perspectives or arguments” (S7) and “avoiding writing errors or structural irregularities” (S4) in terms of report evaluation/refinement/generation. However, they also expressed concerns about a lack of a sense of achievement and engagement. For instance, both S7 and S8 noted that “the written report doesn’t feel like my own achievement—it all belongs to GenAI. Only the musical piece I designed and played by tapping beakers is truly my own work.” Similarly, opinions were divided on contextual awareness and memory. S6 remarked, “Initially, its answers were overly verbose, but after I instructed it to be more concise, its subsequent replies were much clearer.” This experience was directly reflected in the interaction data, for example, “What factors affect a vehicle’s fuel consumption? Brief output!!!!!!” (M-E-55) and “What about the vehicle structure? Brief output!!!!!!!” (M-E-57). Together with S6’s reflection, these excerpts show how the group actively adjusted GenAI’s output style and perceived this strategy as improving the clarity of its subsequent responses. As mentioned earlier, there were cases where GenAI was over-associated with prior context, affecting its responses.

Furthermore, when comparing GenAI-based STEAM modules to traditional courses, students universally praised the richness and extensiveness of interdisciplinary knowledge provided by GenAI, which aligns with the overwhelming dominance of “STEAM knowledge/information” in student–GenAI interaction data. However, upon closer inspection, interactions were often limited to superficial inquiries about basic concepts—neither delving into the core principles nor establishing meaningful connections with the real-world. As S4 noted, “These topics don’t seem strongly connected to the systematic learning in traditional courses.” Additionally, three students explicitly preferred traditional courses. This phenomenon is closely tied to the observation that interaction purposes were overwhelmingly dominated by information requests, with very few review requests.

***Module differences:*** Satisfaction was slightly higher in the M-E module (M = 4.65, SD = 0.43) than in the M-A module (M = 4.54, SD = 0.50). Compared with M-E, most students entered M-A with higher satisfaction or maintained relatively high levels throughout the learning process, possibly due to positive experiences accumulated in the preceding M-E module. As S10 noted, “Although GenAI made minor errors in the M-A module, its impressive performance outweighed these shortcomings.” This process-related pattern does not contradict the slightly higher mean satisfaction for M-E, as retrospective ratings may have been influenced by perceived GenAI-task fit. In module comparisons, seven students considered GenAI more suitable for M-E, one favored M-A, and four reported no difference. Moreover, students’ overall satisfaction was higher than their satisfaction ratings for either individual module. This may be because module-specific ratings were more sensitive to students’ experiences with particular tasks and disciplinary content, whereas the overall rating captured their broader appreciation of GenAI-supported STEAM learning.

Students’ trust in GenAI also varied by module: seven reported no difference, four favored M-E, and one preferred M-A. Preference for M-E was mainly attributed to the view that “there are inherent differences between humans and GenAI in the domains of rationality and sensibility”. GenAI was perceived as better suited to providing objective, factual, and verifiable information in science-focused tasks such as M-E. For example, G2 asked how different engine characteristics and vehicle speeds affect fuel consumption (M-E-35 & M-E-36) and checked GenAI’s science-based responses against prior studies and publicly available data. In contrast, “humanities-focused subjects such as music require creativity and abstract thinking, which rely on uniquely human emotions and imagination—qualities that GenAI struggles to replicate or answer perfectly” (S3, S6). For example, in M-A, G4 and G6 asked GenAI to incorporate the golden ratio to “design a 30 s piece of harmonious music” (M-A-82 & M-A-137). GenAI suggested how to arrange musical climaxes, sections, rhythm, and melody according to the golden ratio, but these suggestions relied more on aesthetic judgment and were less objectively verifiable. This may explain why GenAI appeared more trustworthy in M-E than in M-A.

In contrast to trust, no module differences were reported in GenAI utility. All students reported that GenAI played similar roles across two modules.

## 5. Discussion

This study explores the implementation of GenAI-based STEAM learning environments for K-12 education. Focusing on middle school students, our study examines their interactions with and perceptions of GenAI across mathematics-arts and mathematics-engineering modules. Five key findings emerge from this exploratory study, each meriting further discussion.

First, the students working in groups prioritized STEAM knowledge acquisition, favored exact copying of instructions, demonstrated the highest engagement with direct retrieval/review prompts, and primarily engaged for information retrieval purposes, while showing limited interactions involving review, verification, evaluation, and reflection. This finding resonates with prior research showing that students demonstrate enthusiasm for quickly retrieving and acquiring broad foundational concepts or knowledge but show little inclination for in-depth verification, evaluation, or reflection (e.g., Chen et al., 2025). For instance, Güner and Er (2025) observed that students tend to favor exact copying of instructions, showing limited summarization and spontaneous inquiry.

This pattern may reflect the demands of integrated STEAM learning, where students are expected to work across diverse STEAM knowledge within constrained timeframes (Cai et al., 2024). GenAI can efficiently support this need by providing quick access to STEAM knowledge/information. However, when used mainly as an information-retrieval tool, it may limit deeper cognitive engagement. The relative scarcity of verification, evaluation, and reflection (Güner & Er, 2025; B. Ma et al., 2024) also appeared in this study, echoing concerns that students may accept GenAI outputs without sufficiently questioning, comparing, or improving them.

Accordingly, STEAM teachers could embed verification and revision directly into task requirements. For example, each group could be required to verify at least one key GenAI output using textbooks, reliable websites, or empirical data; compare evidence across sources; justify whether they accept, question, or reject the output; and then revise their ideas through follow-up prompts. In this way, an information retrieval–evidence verification–comparative judgment–revision–re-engagement cycle could be incorporated into stages such as optimization and reflection or team execution and adaptive implementation. This would help ensure that information retrieval leads to evaluation, refinement, and further inquiry rather than becoming the endpoint of interaction.

The frequent exact copying of instructions also suggests the need to support students’ transition from passive use of given prompts to more autonomous and dialogic interaction with GenAI. Prior research indicates that immediate instructor-guided demonstrations can notably reduce this tendency, but this effect may weaken once support is removed (Güner & Er, 2025). Thus, rather than simply providing or withdrawing scaffolds, teachers could use faded prompt scaffolding: teachers may first model complete prompts and explain their components, then provide only task goals or prompt frames for students to complete, and finally require students to formulate, test, and revise prompts independently. Even when instructor-provided prompts are used, students could be required to make at least one personalized modification or self-initiated follow-up and explain their rationale. Such gradual fading may prevent scaffolds from becoming fixed scripts to be copied while preserving necessary support. Achieving an optimal equilibrium between technological empowerment and educational essence—ensuring curricular flexibility (Ruiz-Rojas et al., 2023) and adaptable teacher roles—merits further research.

Second, the students in this study reported high overall satisfaction with GenAI, showing two main patterns: gradual improvement and fluctuation. This finding is consistent with prior studies reporting generally positive student experiences with GenAI-supported learning (e.g., El Fathi et al., 2025; Sun et al., 2024; Tang et al., 2025).

The gradual improvement in satisfaction may be partly related to both increasing familiarity and the novelty effect (Miguel-Alonso et al., 2024). At the initial stage, students may be attracted by GenAI’s impressive and novel capabilities, which can quickly enhance satisfaction. As familiarity increases, however, satisfaction may plateau if students continue to use GenAI in limited or repetitive ways. This suggests that the sustained educational value of GenAI depends not only on students’ positive first impressions, but also on whether they are supported in expanding the depth and diversity of use. Thus, teachers could progressively introduce new GenAI functions and more challenging application contexts, encouraging students to apply GenAI across varied STEAM tasks rather than remaining within routine retrieval-based uses.

Third, the students exhibited high levels of trust but demonstrated limited critical evaluation of GenAI outputs in these GenAI-based learning environments. They primarily relied on prior knowledge and subjective perceptions to assess GenAI outputs, typically detecting obvious or superficial errors only. This pattern aligns with the findings of Dahlkemper et al. (2023), which further demonstrate that the effect is particularly pronounced among students with lower self-assessed expertise. These students often equated “plausible-sounding” answers with “correct” ones, and this cognitive bias was closely linked to an inherent flaw of GenAI—creating “the illusion of competence by avoiding obvious mistakes while still being wrong”. While GenAI’s linguistic quality has reached an unparalleled level, its scientific accuracy remains questionable to varying degrees (e.g., Shoufan, 2023; Wardat et al., 2023). Our study extends these findings, showing that when GenAI states it has “read numerous websites” or “integrated multiple sources”, it appeared to enhance students’ perceived authority of its answers, making them seem less inclined to critique their scientific validity. Notably, only a very small number of students recognized that aggregated information may contain errors. When encountering specialized content beyond their current knowledge base, the students appeared more easily intimidated by GenAI’s superficial authority (Dwivedi et al., 2023).

This phenomenon poses a unique challenge, as STEAM projects often require integrating diverse knowledge and multidisciplinary approaches, making GenAI’s involvement more complex and necessitating more rigorous oversight mechanisms, as well as higher demands on students’ critical thinking. Therefore, educators are recommended to develop specialized training modules (see Singh et al., 2023) to systematically teach students how to evaluate GenAI outputs based on course knowledge or other accessible resources, including identifying potential errors and verifying information sources. Structured reflection sessions should also be emphasized in STEAM projects, encouraging students to validate GenAI information from multiple perspectives.

Fourth, while the participants perceived various roles of GenAI and generally assigned positive or neutral evaluations, they lacked a sense of engagement and derived minimal sense of achievement. Current literature suggests this phenomenon stems from students’ predominant conceptualization of GenAI as either (1) a tool (Min et al., 2025) or (2) a secondary information source (Shoufan, 2023), rather than as a teacher or colleague. When GenAI is regarded as a mere “tool”, interactions tend to be brief and verification-focused, involving information retrieval to satisfy specific curiosities. This interpretation is supported by our finding: the only two roles recognized by all students—information retrieval/integration assistant and slide generation—were both highly instrumental.

In addition, very few students in our study recognized the role of simulated human–machine interaction perception and failed to fully grasp the distinction between GenAI and traditional tools (e.g., Banda & Nzabahimana, 2023; Santilli et al., 2025), which further supports the view that they predominantly perceived it as a tool. Therefore, educators are recommended to consciously guide students to shift their perception of GenAI’s role—from passive “tool users” to active “intelligent collaborative partners”. Another possible explanation lies in the inherent technological limitations of GenAI. Unlike Augmented Reality (AR) (e.g., S. Li et al., 2025) or Virtual Reality (e.g., Boel et al., 2025), which facilitate embodied learning through immersive learning experiences, GenAI’s purely algorithmic content generation, to some extent, diminishes immersion and engagement in the learning process. Future research may seek breakthroughs through multimodal integration: exploring hybrid “GenAI+AR” models that incorporate voice interaction, 3D visualization, and other features, leveraging AR’s immersive interfaces to compensate for the limitations of “flat” interactions.

Fifth, although the overall group-level interaction counts were relatively similar across the two modules, the interaction patterns and perceptions differed in several aspects. The M-A module elicited more spontaneous inquiry awareness, direct retrieval/review prompts, and review requests, whereas the M-E module fostered more optimization engagement. Satisfaction was slightly higher in M-E; most students reported no module difference in trust, but among those who did, more favored M-E. No module difference was found in perceived utility. The similarity in interaction counts and the divergence in perceptions are not necessarily contradictory. In both modules, GenAI use was embedded within predefined learning processes, so the overall amount of interaction was partly constrained by task design and scheduled opportunities for GenAI use. Moreover, satisfaction and trust may depend more on how students experienced, accepted, and evaluated GenAI outputs than on interaction frequency alone.

These differences may be related to the epistemological characteristics of the two disciplinary contexts. Engineering-oriented tasks often involve more clearly defined problems, objective constraints, and verifiable criteria, which may align with GenAI’s capacity to generate structured and comparatively stable responses. In contrast, arts-oriented tasks are more open-ended and rely more heavily on subjective expression, originality, and aesthetic judgment (U. Lee et al., 2024). As a result, GenAI outputs in M-A may have been harder for students to evaluate or accept, even when they were useful. This may help explain why M-E received slightly more favorable satisfaction and trust.

These findings suggest that GenAI should not necessarily play the same role across STEAM contexts. In more solution-oriented tasks, teachers could guide students to compare, justify, and optimize alternative GenAI-generated solutions. In more open-ended creative tasks, students should be supported in developing their own criteria for evaluating, selecting, and revising GenAI outputs. Such differentiated use may allow GenAI to complement, rather than replace, the forms of reasoning and judgment central to learning in different disciplinary contexts.

In summary, this exploratory study offers two contributions to the field. First, it shifts from macro-level evaluations to a micro-level, student-centered analysis of human–GenAI interaction, proposing a three-perspective (“Student,” “Student–GenAI,” and “GenAI”) and four-sub-dimension (interaction content, student standpoint, prompt type, and prompt purpose) framework, with preliminary empirical support for its applicability. Second, it extends GenAI research into STEAM—especially the underexplored arts—showing GenAI’s potential in arts education and broadening focus beyond STEM. Comparisons between mathematics-engineering and mathematics-arts modules indicate that student interactions with GenAI and perceptions regarding GenAI use vary by disciplinary and task contexts, underscoring the need to interpret GenAI’s affordances and limits contextually. These contributions warrant caution due to the small sample size.

## 6. Limitations and Future Studies

While this exploratory study offers valuable insights into students’ interaction with GenAI in STEAM learning, several limitations should be acknowledged.

First, the small and gender-imbalanced sample, laboratory-based setting, and absence of a control or comparison group limit the generalizability and causal interpretation of the findings. The sample included nine boys and three girls, which is important because gender and group gender composition may shape students’ participation and interaction patterns in STEAM learning (L. Ma et al., 2022). Moreover, without a traditional-instruction or non-GenAI comparison group, this study cannot determine the relative effectiveness of GenAI-supported STEAM learning. Future research should employ larger and more gender-balanced samples in authentic classroom settings, include appropriate comparison groups, and explicitly examine the effects of gender and group composition.

Second, several implementation features may have influenced the observed interaction patterns. The study examined only two modules, Mathematics-Engineering and Mathematics-Art, which were delivered sequentially by different instructors. Thus, module differences may have been confounded with instructor effects or module order. In addition, teaching assistants provided on-demand support for GenAI prompt formulation, which may have shaped students’ prompting practices. Future studies should counterbalance module order, use the same instructor across modules where feasible, and standardize, document, or systematically vary prompting support. They could also extend the design to other interdisciplinary configurations, such as Mathematics-Science, Mathematics-Technology, or integrated Mathematics-Science-Engineering modules.

Third, the short duration of the intervention limits conclusions about the stability of students’ interaction patterns and perceptions. Because the study involved only two short-term STEAM modules, the findings may reflect initial or novelty-driven responses rather than sustained forms of GenAI use. Longitudinal research is needed to examine how students’ interactions with GenAI and perceptions of its role over time. In addition, because the research team was involved in both the implementation and subsequent interviews, students’ self-reported perceptions may have been affected by demand characteristics. Future research could reduce this risk by separating instructional and data-collection roles.

Fourth, the measurement of trust was limited. Although open-ended responses offered qualitative insights, the single retrospective rating item captured only a broad judgment of trust and did not fully represent the multidimensional nature of the construct (Kohn et al., 2021). Future research could incorporate validated multi-item trust measures.

Last but not least, the proposed interaction analysis framework requires further empirical validation across diverse educational contexts. Future studies should apply and refine the framework in different learning environments, subject domains, age groups, and levels of GenAI integration to assess its adaptability and explanatory power. In addition, future studies could incorporate evidence of students’ cognitive processes, thereby enabling a more comprehensive understanding of student–GenAI interactions and how learning unfolds in GenAI-supported STEAM learning environments. Such efforts would help establish a more robust theoretical foundation for GenAI-supported STEAM education.

## Figures and Tables

**Table 1:** Analytical framework for student–GenAI interaction.

Sub-Dimension	Category and Explanation	
Interaction Content	STEAM knowledge/information	**Mathematical knowledge/information**
		**Music knowledge/information**
		**Engineering knowledge/information**
	Inquiry plan	**Variables**
		**Process creation**
		**Process modification**
	Problem-solving process	**Data collection**
		**Mathematical model construction and solving**
	Analysis and results	**Result verification**
		**Result interpretation**
	Evaluation and reflection	**Optimization suggestions**
		**Future inquiry directions**
	Presentation and display	**Outcomes organization**
		**Presentation format**
Standpoint	**Exact copy of instruction**	
	**Summary of instruction**	
	**Spontaneous inquiry**	
	**Exact copy of GenAI’s recommendations**	
	**Others**	
Prompt Type	**Direct retrieval/review prompts**: Direct or preliminary information retrieval or review requests.	
	Optimization prompts	**Context optimization prompts**: Supplementing the problem context; asking follow-up questions to obtain further information.
		**Solution optimization prompts**: Requesting simplification of GenAI’s responses or correcting errors in GenAI’s answers; repeating previous prompts to verify solutions.
	**Non-functional scope prompts**: Prompts beyond the functional scope of GenAI.	
	**Others**: Emotional interactions like encouragement or dissatisfaction, or unclear meanings.	
Prompt Purpose	**Information request**: Requesting specific information from GenAI without proposing personal ideas or perspectives.	
	**Review request**: Sharing personal perspectives with GenAI and requesting review and feedback.	
	**Others**: Emotional expressions or unknown purposes.	

**Table 2:** Interaction content (*N* = 370).

Category		Topic		Total
		M-E	M-A	
STEAM knowledge/information	Mathematical knowledge/information	13 (7.1%)	44 (23.7%)	57 (15.4%)
	Music knowledge/information	0 (0.0%)	64 (34.4%)	64 (17.3%)
	Engineering knowledge/information	68 (37.0%)	0 (0.0%)	68 (18.4%)
Inquiry plan	Variables	23 (12.5%)	11 (5.9%)	34 (9.2%)
	Process creation	12 (6.5%)	7 (3.8%)	19 (5.1%)
	Process modification	0 (0.0%)	5 (2.7%)	5 (1.4%)
Problem-solving process	Data collection	22 (12.0%)	34 (18.3%)	56 (15.1%)
	Mathematical model construction and solving	21 (11.4%)	15 (8.1%)	36 (9.7%)
Analysis and results	Result verification	0 (0.0%)	0 (0.0%)	0 (0.0%)
	Result interpretation	12 (6.5%)	4 (2.2%)	16 (4.3%)
Evaluation and reflection	Optimization suggestions	2 (1.1%)	0 (0.0%)	2 (0.5%)
	Future inquiry directions	0 (0.0%)	0 (0.0%)	0 (0.0%)
Presentation and display	Outcomes organization	4 (2.2%)	2 (1.1%)	6 (1.6%)
	Presentation format	7 (3.8%)	0 (0.0%)	7 (1.9%)
**Total**		**184 (100.0%)**	**186 (100.0%)**	**370 (100.0%)**

**Table 3:** Student standpoint (*N* = 310), prompt type (*N* = 315), and prompt purpose (*N* = 310).

Sub-Dimension	Category		Topic		Total
			M-E	M-A	
Student Standpoint	Exact copy of instruction		80 (47.6%)	66 (46.5%)	146 (47.1%)
	Summary of instruction		44 ^a^ (26.2%)	22 ^a^ (15.5%)	66 (21.3%)
	Spontaneous inquiry		39 (23.2%)	50 (35.2%)	89 (28.7%)
	Exact copy of GenAI’s recommendations		5 (3.0%)	2 (1.4%)	7 (2.3%)
	Others		0 (0.0%)	2 (1.4%)	2 (0.6%)
	**Total**		168 (100.0%)	142 (100.0%)	310 (100.0%)
Prompt Type	Direct retrieval/review prompts		98 ^a^ (56.6%)	105 (73.9%)	203 (64.4%)
	Optimization prompts	Context optimization prompts	58 (33.5%)	28 (19.7%)	86 (27.3%)
		Solution optimization prompts	12 (6.9%)	6 (4.2%)	18 (5.7%)
	Non-functional scope prompts		5 (2.9%)	1 ^a^ (0.7%)	6 (1.9%)
	Others		0 (0.0%)	2 (1.4%)	2 (0.6%)
	**Total**		173 (100.0%)	142 (100.0%)	315 (100.0%)
Prompt Purpose	Information request		168 ^a^ (100.0%)	135 (95.1%)	303 (97.7%)
	Review request		0 (0.0%)	5 (3.5%)	5 (1.6%)
	Others		0 (0.0%)	2 (1.4%)	2 (0.6%)
	**Total**		168 (100.0%)	142 (100.0%)	310 (100.0%)

Note: ^a^ refers to including one request with a misinterpretation.

**Table 4:** GenAI utility.

Role	Count	Students	Evaluation
Information retrieval/integration assistant (including web links)	12	S1–S12	Positive
Slide generation	12	S1–S12	Positive
Research plan evaluation/refinement/generation	6	S1, S2, S4, S7, S8, S11	Neutral
Contextual awareness and memory	4	S1, S2, S6, S12	Neutral
Research report evaluation/refinement/generation	3	S3, S4, S7	Neutral
Problem analysis and decision support	2	S1, S11	Neutral
Wording polishing	2	S3, S4	Positive
Simulated human–machine interaction perception	1	S1	Positive

## Data Availability

The datasets generated during and/or analysed during the current study are not publicly available due to privacy considerations concerning the minor participants but are available from the corresponding author on reasonable request.

## Appendix A. Interview Protocol

**Sub-Dimension**	**No.**	**Question**
GenAI user satisfaction	Q1	How satisfied are you with using GenAI in STEAM learning environments? (Rate on a scale of 1-5, where 1 = least satisfied, 5 = most satisfied) Why?
	Q2	How satisfied are you with using GenAI in the M-E and the M-A modules, respectively? (Rate on a scale of 1-5, where 1 = least satisfied, 5 = most satisfied) Why?
	Q3	How did your satisfaction with using GenAI change during the M-E and M-A modules? Please describe any changes you experienced and the reasons for them. Were there any differences between the two modules? (You may refer to your emotion graph to help recall your experiences.)
Trust level	Q5	How much do you trust the information provided by GenAI? (Rate on a scale of 1-5, where 1 = least trust, 5 = most trust) Why?
	Q6	Does your level of trust in the information provided by GenAI vary across different module topics? Why?
	Q7	After receiving a response from GenAI, how do you process the information?
GenAI utility	Q8	In GenAI-based STEAM learning environments, what roles do you think GenAI plays? Please provide examples and give an overall evaluation (positive, neutral, or negative) of its impact.
	Q9	Do you think the role of GenAI differs across module topics?
	Q10	Do you prefer traditional courses or GenAI-based STEAM learning environments? Why?
Others	Q11	What do you think are the advantages/disadvantages of GenAI-based STEAM learning environments?
	Q12	Throughout this course series, which work (your own or others’) was your favorite? What left the deepest impression on you, and why?
	Q13	Do you have any suggestions for improving this course series?

## Appendix B. Coding Examples

**Sub-Dimension**	**Category**		**Explanation**	**Coding Example**
Interaction Content	STEAM knowledge/information	Mathematical knowledge/information	Involving mathematical concepts, formulas, theorems, calculation methods, etc.	M-A-44: The significance and application of the golden ratio.
		Music knowledge/information	Involving music theory, pitch, rhythm, harmony, musical instruments, etc.	M-A-82: How to compose a 30 s music piece.
		Engineering knowledge/information	Involving engineering design, system architecture, materials science, mechanical principles, etc.	M-E-38: Types of vehicle drivetrains.
	Inquiry plan	Variables	Factors or parameters required during the inquiry process	M-E-06: Factors affecting automobile fuel consumption.
		Process creation	Designing and establishing inquiry steps and methods	M-E-151: Research proposal: The Impact of Frontal Area on Fuel Consumption.
		Process modification	Adjusting and optimizing the original plan based on feedback or results during the inquiry process	M-A-54: See Figure 3b.
	Problem-solving process	Data collection	Collecting relevant data from different sources	M-E-40: Which websites can be used to search for vehicle fuel consumption data?
		Mathematical model construction and solving	Building a mathematical model based on the collected data and solving it using mathematical methods.	M-E-13: Plot the function graph.
	Analysis and results	Result verification	Verifying the correctness of the model or hypothesis through experiments, simulations, or other methods.	/
		Result interpretation	Interpreting and explaining the analysis results to draw conclusions.	M-E-75: Energy-efficient car design report: Meeting the following three criteria—reducing mass, footprint, and frontal area.
	Evaluation and reflection	Optimization suggestions	Proposing improvement plans or optimization suggestions based on the inquiry results.	M-E-165: Provide optimization suggestions based on the report.
		Future inquiry directions	Identifying future research directions based on the limitations of the current study or new discoveries.	/
	Presentation and display	Outcomes organization	Organizing and summarizing research findings into systematic reports or papers.	M-A-168: Summarize the core content.
		Presentation format	Presentation formats for results, such as charts, presentations, videos, interactive interfaces, etc.	M-A-167: Generate a PPT based on this document.
Standpoint	Exact copy of instruction		Directly copy-pasting entire task instructions	M-A-59: Spearman’s rank correlation coefficient.
	Summary of instruction		Summarizing/paraphrasing task instructions	M-E-22: How to mathematically compare the correlation between different factors and vehicle fuel consumption?
	Spontaneous inquiry		Proposing inquiry prompts related to the task independently.	M-E-149: Why do streamlined car bodies have less air resistance?
	Exact copy of GenAI’s recommendations		Directly using GenAI-generated associated prompts.	M-E-4: How to consider fuel efficiency when buying a car?
	Others		Ambiguous prompts that cannot be clearly positioned.	M-E-111: .
Prompt Type	Direct retrieval/review prompt		Direct or preliminary information retrieval or review requests.	M-A-10: Is all harmonious music related to the golden ratio?
	Optimization prompt	Context optimization prompt	Supplementing the problem context; asking follow-up questions to obtain further information.	M-E-11: List more data.
		Solution optimization prompt	Requesting simplification of GenAI’s responses or correcting errors in GenAI’s answers; repeating previous prompts to verify solutions.	M-E-84: What are the basic structures of a car? (Concise)
	Non-functional scope prompt		Prompts beyond the functional scope of GenAI, such as requesting text-based GenAI to output visual representations.	M-E-89: Automobile structure diagram.
	Others		Emotional interactions like encouragement or dissatisfaction, or unclear meanings.	M-E-7: Are you stupid?
Prompt Purpose	Information request		Requesting specific information from GenAI without proposing personal ideas or perspectives.	M-A-21: Simplified musical notation for Wang Leehom’s “Big City, Small Love”
	Review request		Sharing personal perspectives with GenAI and requesting review and feedback.	Ditto M-A-54.
	Others		Emotional expressions or unknown purposes.	Ditto M-E-7.
